# Supplementary material for: Navigating in Virtual Environments: Does a Map or a Map-Based Description Presented Beforehand Help?
Source: Brain Sci. 2021 Jun 10;11(6):773. doi: 10.3390/brainsci11060773 (PMC8230476; doi:10.3390/brainsci11060773)
Supplement: Supplementary file 1 [file brainsci-11-00773-s001.zip › brainsci-1255526-supplementary.pdf]

Table S1. Means and standard deviations (in brackets) of log-transformed scores of route retracing (errors), pointing (errors) and path drawing (accuracy) by group.

	Map before Navigation Group	Description before Navigation Group	Only Navigation Group
Route retracing task	0.70 (0.26)	0.64 (0.19)	0.70 (0.16)
Pointing task	1.11 (0.76)	1.36 (0.66)	1.66 (0.50)
Path drawing task	1.24 (0.08)	1.00 (0.26)	1.06 (0.15)

Table S2. Correlations between individual difference measures and recall task performance in the "map before navigation" group.

	Route Retracing Task	Pointing Task	Path Drawing Task
Corsi Task (backward)	0.130	0.037	−0.065
Digit Span Task (backward)	−0.136	−0.170	−0.074
Mental Rotations Test	0.032	−0.310	−0.089
Spatial Anxiety Scale	−0.085	−0.231	0.047
SDSR—Sense of direction—preference for survey mode	−0.142	0.003	−0.078
SDSR—Knowledge and use of cardinal points	−0.117	−0.143	−0.194
SDSR—preference for landmark-route mode	−0.090	0.195	−0.087

Table S3. Correlations between individual difference measures and recall task performance in the "description before navigation" group.

	Route Retracing Task	Pointing Task	Path Drawing Task
Corsi Task (backward)	−0.137	0.053	0.102
Digit Span Task (backward)	−0.070	−0.074	0.076
Mental Rotations Test	−0.216	0.061	0.182
Spatial Anxiety Scale	0.027	−0.307	0.040
SDSR—Sense of direction—preference for survey mode	−0.270	0.001	−0.193
SDSR—Knowledge and use of cardinal points	−0.037	0.125	0.046
SDSR—preference for landmark-route mode	−0.196	−0.100	−0.449

Table S4. Correlations between individual difference measures and recall task performance in the "only navigation" group.

	Route Retracing Task	Pointing Task	Path Drawing Task
Corsi Task (backward)	−0.214	0.025	−0.258
Digit Span Task (backward)	**−0.464**	0.060	−0.112
Mental Rotations Test	−0.146	0.079	−0.184
Spatial Anxiety Scale	0.301	−0.030	−0.133
SDSR—Sense of direction—preference for survey mode	−0.166	−0.278	−0.230
SDSR—Knowledge and use of cardinal points	−0.252	−0.173	−0.157
SDSR—preference for landmark-route mode	−0.277	−0.308 *	−0.003

The values in bold express medium-high degree of relations.
